# Bleeding in Hepatic Surgery: Sorting through Methods to Prevent It

**DOI:** 10.1155/2012/169351

**Published:** 2012-11-18

**Authors:** Fabrizio Romano, Mattia Garancini, Fabio Uggeri, Luca Degrate, Luca Nespoli, Luca Gianotti, Angelo Nespoli, Franco Uggeri

**Affiliations:** Unit of Hepatobiliary and Pancreatic Surgery, Department of Surgery, San Gerardo Hospital, University of Milan-Bicocca, Via Donizetti 106, 20052 Monza, Italy

## Abstract

Liver resections are demanding operations which can have life threatening complications although they are performed by experienced liver surgeons. The parameter “Blood Loss” has a central role in liver surgery, and different strategies to minimize it are a key to improve results. Moreover, recently, new technologies are applied in the field of liver surgery, having one goal: safer and easier liver operations. The aim of this paper is to review the different principal solutions to the problem of blood loss in hepatic surgery, focusing on technical aspects of new devices.

## 1. Introduction

Liver resection is considered the treatment of choice for liver tumours. Despite standardized techniques and technological advancing for liver resections, an intraoperative haemorrhage rate ranging from 700 to 1200 mL is reported with a postoperative morbidity rate ranging from 23% to 46% and a surgical death rate ranging from 4% to 5% [1–6].

The parameter “*Blood loss*” has a central role in liver surgery and different strategies to minimize it are a key to improve these results. Bleeding has to be considered a major concern for the hepatic surgeon because of several reasons. At first, it is certainly the major intraoperative surgical complication and cause of death and historically one of the major postoperative complication together with bile leaks and hepatic failure [5–9].

Besides, a high intraoperative blood loss is associated with a higher rate of postoperative complication and shorter long-term survival [10–13]. Furthermore, it is associated with an extensive use of vessel occlusion techniques, directly correlated with higher risk of postoperative hepatic failure. Last, a higher value of intraoperative blood loss is associated with a higher rate of perioperative transfusions; and host immunosuppression associated with transfusions with a dose-related relationship is correlated with a higher rate of complication (in particular infections) and recurrence of malignancies in neoplastic patients [11, 12, 14–21]. In order to reduce transfusions, hepatic surgeon has also not to misinterpret postoperative fluctuations of blood parameter: Torzilli et al. demonstrated that haemoglobin rate and haematocrit after liver resection show a steady and significant decrease until the third postoperative day and then an increase, so this situation has to be explained as physiological and does not justifies blood administration [22].

## 2. How Can We Reduce Bleeding in Liver Surgery?

This study is based on the literature information and our own experience.

The aim of the study is to investigate the principal solutions to the problem of high blood loss in hepatic resection.

### 2.1. The Role of the Surgeon

Most blood loss during liver resection occurs during parenchymal transection. Hepatic surgeon has different ways to control bleeding.

#### 2.1.1. Vessel Occlusion Techniques

Those techniques are based on the idea that to limit the blood flow through the liver during parenchymal transection can reduce the haemorrhage. Although various forms and modified techniques of vascular control have been practiced, there are basically two main strategies; inflow vascular occlusion and total vascular exclusion [23, 24]. Inflow vascular occlusions are techniques that limit anterograde blood flow with the clamping of all the triad of the hepato-duodenal ligament (*Pringle's manoeuvre*), only of the vascular pedicles (selective clamping of the portal vein and the hepatic artery or *bismuth technique*) or *intravascular portal clamping*. During Pringle's maneuvre, the hepatoduodenal ligament is encircled with a tape, and then a vascular clamp or tourniquet is applied until the pulse in the hepatic artery disappears distally. The PM has relatively little general haemodynamic effect and no specific anaesthetic management is required. However, bleeding can still occur from the backflow from the hepatic veins and from the liver transection plane during unclamping. The other concern is the ischaemic-reperfusion injury to the liver parenchyma, especially in patients with underlying liver diseases [25]. The continuous Pringle manoeuvre (CPM) can be safely applied to the normal liver under normothermic conditions for up to 60 minutes and up to 30 minutes in pathological (fatty or cirrhotic) livers, although much longer durations of continuous clamping 127 minutes in normal livers and 100 minutes in pathological livers have been reported to be safe [26, 27]. One way to extend the duration of clamping and to reduce ischaemia to the remnant liver is by the intermittent Pringle manoeuvre (IPM). It involves periods of inflow clamping that last for 15–20 minutes followed by periods of unclamping for five minutes (mode 15/5 or 20/5), or five minutes clamping followed by one minute unclamping (mode 5/1) [28, 29]. IPM permits a doubling of the ischaemia time, when compared with CPM, and the total clamping time can be extended to 120 minutes in normal livers and 60 minutes in pathological livers. The disadvantage of IPM is that bleeding occurs from the liver transaction surface during the unclamping period and, thus, the overall transection time is prolonged as more time is spent in achieving haemostasis. Belghiti et al. [28] revealed that there was no significant difference in total blood loss or volume of blood transfusion between CPM and IPM (mode 15/5). However, they noticed that pathological livers tolerated CPM poorly.

A newer perspective on inflow occlusion comes from the concept of ischaemic preconditioning (IP). It refers to an endogenous self-protective mechanism by which a short period of ischaemia followed by a brief period of reperfusion produces a state of protection against subsequent sustained ischaemia-reperfusion injury [30, 31]. The IP is performed with ten minutes of ischaemia followed by ten minutes of reperfusion before liver transaction with CPM [32]. Hemihepatic clamping (half-Pringle manoeuvre) interrupts the arterial and portal inflow selectively to the right or left liver lobe that is to be resected [33, 34]. It can be performed with or without prior hilar dissection. It can also be combined with simultaneous occlusion of the ipsilateral major hepatic vein. The advantage of this technique is that it avoids ischaemia in the remnant liver, avoids splanchnic congestion, and allows clear demarcation of the resection margin. The disadvantage is that bleeding from the parenchymal cut surface can occur from the nonoccluded liver lobe.

Segmental vascular clamping entails the occlusion of the ipsilateral hepatic artery branch and balloon occlusion of the portal branch of a particular segment. The portal branch is identified by intraoperative ultrasound and puncture with a cholangiography needle through which a guide wire and balloon catheter are passed [35, 36]. 

Total vascular exclusion (TVE) combines total inflow and outflow vascular occlusion of the liver, isolating it completely from the systemic circulation. It is done with complete mobilisation of the liver, encircling of the suprahepatic and infrahepatic IVC, application of the Pringle manoeuvre, and then clamping the infrahepatic IVC followed by clamping of the suprahepatic IVC. TVE is associated with significant haemodynamic changes and warrants close invasive and anaesthetic monitoring. Occlusion of the IVC leads to marked reduction of venous return and cardiac output, with a compensatory 80% increase in systemic vascular resistance and 50% increase in heart rate and, thus, not every patient can tolerate it. TVE can be applied to a normal liver for up to 60 minutes and for 30 minutes in a diseased liver. The ischaemic time can be extended when combined with hypothermic perfusion of the liver [37, 38]. Apart from the unpredictable haemodynamic intolerance, postoperative abdominal collections or abscesses and pulmonary complications are more common in TVE, when compared with CPM. 

Inflow occlusion with extraparenchymal control of hepatic veins is a modified way of performing TVE. The main and any accessory right hepatic vein, the common trunk of the middle and left hepatic veins, or the separate trunks of the middle and left hepatic veins (15% of cases) are first dissected-free and looped. It has been reported that the trunks of the major hepatic veins can be safely looped in 90% of patients [39, 40]. The loops can then be tightened or the vessels can be clamped after inflow occlusion is applied, so that the liver lobe is isolated from the systemic circulation without interrupting the caval flow. It can be applied in a continuous or intermittent manner. The maximal ischaemia time is up to 58 minutes under continuous occlusion. This technique is more demanding than TVE, but it can avoid the haemodynamic drawbacks of TVE while at the same time provide almost a bloodless field for liver transection. 

#### 2.1.2. Instruments and Technique for Resections

Although a large part of improvements of these last decades in liver surgery can be correlated to a better knowledge of the surgical hepatic anatomy (Couinaud's segmentation of liver [41]), better monitoring during anaesthesia, and introduction of intraoperative ultrasonography and of other imaging techniques, the choice of surgical technique for sectioning the liver has surely important repercussions on the intervention's outcome.

There are two techniques we could define traditionally: the *finger fracture method* and the *clamp crushing method*. These are the oldest techniques for hepatic transection and are still employed especially by long-experienced surgeons. The use of traditional techniques to isolate bile ducts and vascular pedicles from the surrounding parenchyma provides for employment of clips or sutures for sealing bile ducts and vascular vessels and for other haemostasis techniques to stop haemorrhage from the resection's surface. There are several studies that sustain that traditional methods are still competitive with a new technique based on utilization of special devices [1, 42, 43].

Introduction of new devices for liver dissection surely have an important role, in particular for reduction of intraoperative blood loss. Actually the most important devices useful for liver resection are presented a technical point of view and analysed to find the advantages (A) and the disadvantages (D) correlated to their employment.

*Harmonic Scalpel*, HS (Johnson and Johnson Medical, Ethicon, Cincinnati, OH, USA), also known as “Ultrasonically Activated Scalpel” or “Ultrasonic Coagulation Shears,” this instrument was introduced in the early 1990s. The ultrasound scissors system includes a generator with a foot switch, the reusable handle for the scalpel, and the cutting device with scissors. The scissors are composed by a moveable blade and by a fixed longitudinal blade that vibrates with an ultrasonic frequency of 55,5 kHz (55.500 vibrations per second). HS can simultaneously cut and coagulate causing protein denaturation by destroying the hydrogen bonds in proteins and by generation of heat in vibrating tissue. This generated heat denatures proteins and forms a sticky coagulum that covers the edges of dissection. Although the heat produces no smoke and thermal injury is limited, the depth of marginal necrosis is greater than that incurred by either the water jet or CUSA. The lateral spread of the energy is 500 micrometers. 

A: HS is the only instrument that can simultaneously cut and coagulate (it can coagulate vessel until 2-3 mm of diameter [44]); it is useful on cirrhotic liver [45]; no electricity passes through the patient and there is no smoke production (especially useful in laparoscopic surgery); it can be used in laparoscopic and laparotomic surgery. D: the instrument results in a continuous bleeding risk related to the blind tissue penetration to coagulate vessels hidden into the hepatic parenchyma. Studies demonstrate that HS is not capable of reducing blood loss and operating time compared to traditional techniques [46, 47], cannot coagulate vessel over 2-3 mm of diameter which have to be clipped, and is legated or sealed with other instruments; HS is not easy to use as a blunt dissector and has substantially demonstrated its usefulness only during the resection of the superficial part of liver (2-3 cm) free from large vessels and bile ducts; besides some studies have demonstrated that HS increases the rate of postoperative bile leaks [48, 49], raising the concern that HS may not be effective in sealing bile ducts. The use of HS in liver cirrhosis is controversial. The greatest concern with the use of the harmonic scalpel is the risk of shearing [50]. Slight errors of movement can shear parenchyma without completely coagulating vessels and/or ducts. Moreover, it is expensive (the generator costs US$20.000 and the handle US$250).

#### 2.1.3. Cavitron Ultrasonic Surgical Aspirator, CUSA (Valleylab)

The use in liver surgery of this instrument, also known as ultrasonic dissector, was described for the first time in the literature in 1979 by Hodgson [51]. CUSA is a surgical system in which a pencil-grip surgical hand piece contains a transducer that oscillates longitudinally at 23 kHz and to which a hollow conical titanium tip is attached. The vibrating tip of the instrument causes explosion of cells with a high water content (just like hepatocytes) and fragmentation of parenchyma sparing blood and bile vessel because of their walls prevalently composed by connective cells poor of water but rich of intracellular bonds. The device is equipped by a saline solution irrigation system that cools the hand piece and washes the transection plane and by a constant suction system that removes fragmented bits of tissue and permits excellent visualization. A: CUSA is capable of dissecting, offering excellent visualization useful in particular during nonanatomical resections and approaching the deeper portion of the transaction plane [52, 53]. (1) The instrument allows surgeons to see clearly blood and biliary vessels as they dissect through the liver [54], (2) use of the instrument allows them to avoid prolonged extrahepatic vascular control, and (3) the operation actually takes less time because the vessels are continuously controlled during the dissection and there is little need for a prolonged search for bleeding or biliary vessels after the specimen has been removed.

A previous retrospective study from Fan showed that the ultrasonic dissector resulted in lower blood loss, lower morbidity, and lower mortality compared with the clamp crushing technique [55]. Furthermore, ultrasonic dissection resulted in a wider tumor-free margin because of a more precise transection plane.

D: CUSA cannot coagulate or realize haemostasis and even if some studies sustain it to be capable of reducing intraoperative blood loss, operating time, and duration of vessel occlusion [56], important studies demonstrate that CUSA cannot offer these advantages if compared with traditional techniques; a prospective trial by Rau et al. showed no statistical difference in the reduction of blood loss with the use of CUSA as compared to conventional methods [57]; another trial by Takayama et al. [53], in fact, noted a greater median blood loss. CUSA causes more frequent tumour exposure at the surgical margin than traditional techniques [1] and it is less useful for cirrhotic livers because the associated fibrosis prevents easy removal of hepatocytes [58]; besides, some authors found using CUSA method (compared to clamp crushing method) an increase of venous air embolism without evidence of hemodynamic compromise but with increased risk of paradoxical embolism in cirrhotic patients [59]. Moreover, CUSA should be used in association with other devices which are able to perform hemostasis. The instrument seems cumbersome and complicated to inexperienced operating room personnel. Therefore, it is easy for the instrument to malfunction. The fact that the instrument works by removing a margin of liver tissue makes it, by nature, less attractive for harvesting liver for living-donor transplantation.

#### 2.1.4. Tissuelink Monopolar Floating Ball, TMFB (Floating Ball, TissueLink Medical, Dover, NH, USA)

This new instrument was put on the market in 2002 and it is a linear device that employs radiofrequency (RF) energy focused at the tip to coagulate target tissue. The tip is provided with a low volume (4–6 mL/min) saline solution irrigation that makes easier the conduction of RF in surrounding tissue and cools the tip itself avoiding formation of chars. TMFB can seal vascular and bile structures up to 3 mm in diameter by collagen fusion. These qualities make this device an excellent instrument for achieving haemostasis and in particular for precoagulating (with a painting movement) parenchyma and vessels prior to transection, preventing blood loss. 

Otherwise, continuously heating tissue underneath a cool layer, however, causes a buildup of steam that can result in tissue destruction. The latter phenomenon is known as steam popping [60].

There are two models on the market, the DS3.0 with blunt tip that simply coagulates and the DS3.5-C Dissecting Sealer that is provided with sharp tip that can also dissect. A: the instrument is, in a sense, “friendlier” to most surgeons. In other words, surgeons, who are usually adept at using cautery, can easily understand this mechanism of action and use it accordingly. TMFB can coagulate (and the Dissecting Sealer can also cut) tissues and seals blood and bile ducts up to 3 mm in diameter, is able to reduce blood loss and the recourse to vessel occlusion techniques if compared to traditional techniques [61–63], offers good results also in cirrhotic livers and cystopericystectomy [64], and has a saline irrigation that avoids production of smoke, chars, and sticky coagulum to which the device could stick causing new bleeding when it is moved away. TMFB, used on the cut liver surface after dissection, destroys eventual additional cancer cells at the margin of resection; in order to assure sterile margins, extra tissue destruction at the margins of resection may be desirable for tumor excisions. Otherwise, this could be a disadvantage in case of living-donor liver transplantation. It is available for both laparotomic and laparoscopic surgery and it is quite cheap and compatible with most electrosurgical generator currently available.

 D: TMFB is not able to coagulate vessel over 2-3 mm of diameter which has to be clipped, legated, or sealed with other instruments [65]. Moreover, studies do not demonstrate its efficacy to reduce operating time if compared with traditional techniques [66].

#### 2.1.5. The Aquamantys System

The Aquamantys System employs transcollation technology to simultaneously deliver RF (radiofrequency) energy and saline for haemostatic sealing and coagulation of soft tissue and bone at the surgical site. Transcollation technology is used in a wide variety of surgical procedures, including orthopaedic joint replacement, spinal surgery, orthopaedic trauma, and surgical oncology. Transcollation technology simultaneously integrates RF (radiofrequency) energy and saline to deliver controlled thermal energy to the tissue. This allows the tissue temperature to stay at or below 100°C, the boiling point of water. Unlike conventional electrosurgical devices which operate at high temperatures, transcollation technology does not result in smoke or char formation when put in contact with tissue. Blood vessels contain Type I and Type III collagen within their walls. Heating these collagen fibers causes radial compression, resulting in a decrease in vessel lumen diameter. Using the Aquamantys generator with patented bipolar and monopolar sealers, surgeons can achieve broad tissue-surface haemostasis by applying transcollation technology in a painting motion, or it can be used to spot-treat bleeding vessels. This is capable of sealing structures 3–6 mm in diameter without producing high temperature or excessive charring and eschar. Structures more than 6 mm in diameter should be divided in conventional manner with clips or ties. Constant suction is required to clear the saline used for irrigation.

A: its use is “friendlier” to most surgeons, easy to learn most surgeons are comfortable after 5-6 procedures. It seals blood and bile ducts up to 6 mm in diameter and is able to reduce blood loss and the recourse to vessel occlusion techniques. Moreover, it offers good results also in cirrhotic livers [67] and destroys eventual additional cancer cells at the margin of resection.

D: it is expensive and pace of liver transaction could be low.

#### 2.1.6. Bipolar Vessel Sealing Device, BVSD (LigaSure, Valleylab Inc., Boulder, CO, USA)

The use in liver surgery of this instrument was described for the first time in the literature in 2001 by Horgan [68]. The LigaSure System includes a generator with a foot switch and a clamp-form hand piece that can be used for parenchymal fragmentation and isolation of blood and bile structures just like in clamp crushing technique before application of energy; it employs RF to realize permanent occlusion of vessels or tissue bundle. The LigaSure generator has a Valleylab's Instant Response technology, a feedback-controlled response system that diagnoses the tissue type in the instrument jaws and delivers the appropriate amount of energy to effectively seal the vessel: when the seal cycle is complete, a generator tone is sound, and the output to the handset is automatically discontinued. BVSD is capable of obliterating the lumen of veins and arteries up to 7 mm in diameter by the fusion of elastin and collagen proteins of the vessel walls and that makes BVSB the only safe and real alternative to sutures and clips for sealing vessel [69–71].

A: BVSD coagulates sealing vessels up to 7 mm in diameter with minimal charring, thermal spread, or smoke; it is capable of reducing blood loss and the recourse to vessel occlusion techniques if compared to traditional techniques [8, 72, 73]. A recently published randomized controlled trial demonstrated that the use of LigaSure in combination with a clamp crushing technique resulted in lower blood loss and faster transaction speed in minor liver resections compared with the conventional technique of electric cautery or ligature for controlling vessels in the transection plane [74]. Otherwise, a more recent randomized trial from the same team was not able to show a real difference between the traditional techniques and the LigaSure vessel sealing system [75]. The instrument is available for both laparotomic and laparoscopic surgery [76]. Furthermore, the use of LigaSure System is not correlated with an increase of the rate of postoperative bile leaks and in some studies bile leakage was nihil [77] and that proves its effectiveness in obliterating also bile vessel. D: after the application the coagulated tissue often sticks to the instrument's jaws causing new bleeding when the device is moved away; BVSD seems to be less effective in presence of cirrhosis for two reasons: first the portal hypertension correlated with cirrhosis causes thinning of the dilated portal vein's walls and makes their obliteration less effective; second cirrhosis makes crushing technique difficult and the hepatic tissue between the blades may disperse the power applied causing vessel to bleed; moreover, it seems to be ineffective in cystopericystectomy [78] (even if some surgeons sustain its effectiveness in this surgery [79]).

#### 2.1.7. Water Jet Scalpel: WJS

The WJS was introduced in 1982 by Papachristou [80]. The device consists of a pressure generating pump and a flexible hose connected to the hand piece. The liquid (saline solution) flows at a steady stream and is projected through the nozzle at the tip of the hand piece. The jet hits the liver at the desired line of transection and washes away the parenchyma, leaving the intrahepatic ducts and vessel undamaged, then the vascular and bile structures can be legated and the transection plane coagulated. The tip is reinforced by a suction tube which removes excess fluid, besides splashing is avoided by covering the area of dissection with a transparent sheet or a Petri dish. Compared to the CUSA, the water jet leaves a smoother cut surface and little hepatic degeneration or necrosis at the borders.

A: WJS can dissect, offering excellent visualization, and is effective also in the cirrhotic liver. In the only available prospective randomized trial of water jet in the literature, in which 31 patients underwent liver resection using water jet and another 30 patients underwent liver resection using CUSA, water jet transection reduced blood loss, blood transfusion, and transection time compared with CUSA [81]. Water jet techniques are quite good for dissecting out major hepatic veins when tumors are in proximity. This allows for delineation of hepatic veins, particularly at the junction with the inferior vena cava, and prevents positive margin. 

D: WJS cannot coagulate or realize haemostasis and some studies demonstrate that it cannot achieve a reduction of intraoperative blood loss and operating time if compared with traditional techniques [82, 83]; using this technique is possible in cancerous seeding of the healthy abdominal organs and infection of the operators by hepatic viruses; moreover, in the literature some cases of gas embolism are described using this device [84]. Furthermore, the instrument may be more effective than the CUSA with respect to operating in the presence of cirrhosis. Papachristou and Barters [80] initially reported that the water jet was likely to be ineffective when there is increased fibrotic tissue. Later papers, however, describe successful resections with cirrhosis by using higher jet pressures. Une et al. [81] report that one does not need to use higher water jet pressures to dissect cirrhotic tissue effectively; instead, the same pressures as for normal parenchyma just need to be applied longer. The major concern of surgeons using the water jet is the associated splash. The latter effect is caused by solution bouncing off tissues. Besides, the obvious infectious concerns of the possibility of contaminating operating room personnel, the splash, bring up the notion of the possibility of cancerous seeding. This possibility must be considered in operations for malignancy and one needs to take additional care not to be exposed to the gross tumor during the dissection.

#### 2.1.8. Staplers

Staplers can be used in liver surgery for control of inflow and outflow vessels, or to divide liver parenchyma [85, 86]. The stapler is rarely used as the principal instrument in hepatic resection. The device can add speed to the operation in open or laparoscopic surgery. Its primary use is for achieving control of hepatic vasculature, particularly the hepatic veins. Biliary radicals can be incorporated efficiently into the staple line. Division of the hepatic veins with a stapler as opposed to direct ligation proffers several advantages. First, it eliminates the risk of dissecting the hepatic veins and minimizes the risk of slipped ligature. Furthermore, the stapler simultaneously divides multiple venous branches, especially on the right side, that are too short to allow for a safe, rapid and more traditional ligation.

It is particularly useful in dividing the major trunk of hepatic veins or the middle hepatic vein deep in the transaction. Vascular staplers also can be used to divide the hepatic duct pedicle in the right or left hepatectomy [7]. The procedure starts by dividing the liver capsule by diathermy, the use of a stapler for transection of the liver parenchyma, followed by fracturing the liver tissue with a vascular clamp in a stepwise manner and subsequently divided with an ENDOGia vascular stapler. In a large series of 300 stapler hepatectomies, including 193 major hepatectomies, mortality of 4% and morbidity of 33% were reported which is comparable with conventional liver resection techniques. Vascular control was necessary in only 10% of the series, with an overall median blood loss of 700 mL [87]. Although the technique appears attractive, the financial cost is a serious drawback. One problem associated with the use of a stapler for liver transection is the increased risk of bile leak, since the stapler is not very effective in sealing small bile ducts [88]. Moreover, the surgeon must also be selective in the use of a stapler for the treatment of tumors particularly near the hilum in order to obtain sufficient margin. In case of stapler malfunction, the surgeon should be ready with a backup technique to achieve vein control in case of a sudden hemorrhage.

#### 2.1.9. Habib's Technique

This technique, invented by Habib in 2002, is also known as bloodless hepatectomy technique [10, 89]. Resection is conducted using cooled tip radiofrequency probe that contains a 3 cm exposed tip to coagulate liver resection margins. Once a 2 cm wide coagulative necrosis zone is created by multiple applications of the probes in adjacent zones and at different depths, the division of the parenchyma with a surgical scalpel is possible. Both the remnant liver and the removed specimen have on the margin of resection a portion of necrotic coagulated liver 1 cm thick. 

A: Habib's technique allows hepatic resections with marginal blood loss, without any vessel occlusion technique or intra- or postoperative transfusions. In a preliminary study of 15 cases of mainly segmental or wedge resection reported by Weber et al., the mean blood loss was only 30 ± 10 mL, and no complications such as bile leakage were observed [89]. Another group also reported low blood loss using this technique in liver resection [90]. Haemostasis is obtained only by RF thermal energy: no additional devices like stitches, knots, clips, or fibrin glue are needed [10, 89, 91, 92]; it is effective also in the cirrhotic liver and the 1 cm thick of burned coagulated surface assures margins free from tumour. The technique has the advantage of simplicity compared with the aforementioned transection techniques.

D: Habib's technique cannot be applied near the hilum or the cava vein for fear of damaging this structures and because the blood flow of large vessels subtracts RF energy and involves an incomplete coagulative necrosis [93, 94] (up to now the technique has been experienced only for segmental resection); the 1-cm-thick of burned coagulated layer in the surface involves the loss of part of healthy parenchyma and a higher rate of postoperative abdominal abscesses [92, 95]. Moreover, one potential disadvantage of this technique is the sacrifice of parenchymal tissue in the liver remnant, with a 1 cm wide necrotic tissue at the transection margin, which may be critical in cirrhotic patients who require major liver resection.

#### 2.1.10. Chang's Needle Technique

This technique presented by Chang in 2001 [96] is based on the utilization of a special instrument equipped with an 18 cm straight inner needle with a hook near its top; Chang needle can be applied repeatedly to make overlapping interlocking mattress sutures with N° l silks along the inner side of the division line. After this phase, liver parenchyma can be divided directly by scissors, electrocautery or traditional resection methods applying new suture only for tubular structures of significant size. 

A: Chang's needle technique can be safely used without vascular occlusion, without any other hemostatic technique thus obtaining a reduction in blood trasfusion rates. This method seems to be capable of reducing both intraoperative blood loss and resection time; besides it is surely cheap and is reported to be simple too [43].

D: it cannot be applied if the lesion is too close to inferior cava vein [97].

#### 2.1.11. Gyrus PlasmaKinetic Pulsed Bipolar Coagulation Device

Gyrus (Gyrus Medical Inc., Maple Groves, MN, USA) is a bipolar cautery device which seals the hepatic parenchyma using a combination of pressure and energy that results in the fusion of collagen and elastin in the walls of the hepatic vasculature and bile ducts [98]. The device can reliably seal vessels up to 7 mm in diameter minimizing the amount of blood loss during the transection of the liver. Thermal spread and sticking to tissues is reduced by a cooling period after each pulse as the impedance of the coagulated tissue increased. This instrument has been widely used in previous gynaecological procedures and its use in liver surgery is relatively new. It could be used in a similar manner to the clamp-crush technique to transect hepatic parenchyma. After incising the hepatic capsule with Bovie, the instrument is inserted into the liver in an open manner and bipolar energy is applied as the forceps are slowly closed over the parenchyma. In a recent series, median blood loss rate is compared favourably with those in several large series using the traditional clamp-crush technique [99]. Moreover, blood loss and transfusion rates were comparable with those cited in recent report of alternative parenchymal transaction, as showed by results of Tan et al. [100]. In this study, Gyrus is compared favourably with Harmonic scalpel in terms of bile leakage and the author underlined the concorrential cost of the device. Moreover, it seems to be useful even in case of cirrhotic patients. Corvera et al. [98] have also reported the use of the Gyrus device in cirrhotic livers comparing it to the clamp and crush technique. They evaluated five patients in each group showing similar results between the two groups in terms of operating time, blood loss, and major postoperative complications.

#### 2.1.12. Haemostasis Techniques

Coagulation of vessels over 1 mm of diameter can be achieved by positioning clips or sutures before division, or using devices like LigaSure, TMFB, or HS for their target vessels or staplers for the largest veins. Clips and sutures are used especially during transaction through traditional techniques.

During and after liver's transaction, haemostasis of the vascular structures under 1 mm of diameter is another important concern of the surgeon: firstly because the continuous bleeding from the little vessels in the parenchyma represents a considerable part of intraoperative blood loss, and secondly because it makes hard for the surgeon to visualize the surgical field. The stop of tearing small vessels that causes oozing from the cut surface can be achieved with normal monopolar or bipolar electrocoagulator, better if equipped with saline irrigation that makes them less traumatic and avoids formation of sticky coagulum. An alternative is represented by employment of Argon Beam Coagulator or TMFB that probably is the best device for stopping tearing of small vessels on the cut surface of the liver.

After the resection, another two precautions can be taken: application of mattress sutures for providing a mechanical compression of the bare surface and application of biological glue for realizing complete haemostasis through a chemical/biological action.

#### 2.1.13. Choice of Surgical Strategy

The choice of surgical strategy is based on the preoperative evaluation and on the now indispensable intraoperative ultrasonography (IOUS); in fact several studies have demonstrated that the IOUS is capable of changing surgical strategy in over 40% of cases finding new lesions or diagnosing as inoperable lesions those which were thought as operable at the previous evaluation [101–104]. The kind of surgical strategy chosen for the intervention on the base of the effects strongly influences the operative outcome and the amount of operative blood loss. The most considerable aspect is the amplitude of the resection: a large resection like a right hemihepatectomy (or another typical resection) involves a higher bleeding and a risk of complications. From this point of view, the choice of segmental or wedge limited resections, when they are possible in respect of radical oncology standards, has to be considered as the best option [105, 106]. Usual surgical margins for removal of liver tumours are 1 cm of healthy parenchyma surrounding the lesion. Kokudo et al. in 2002 demonstrated that for colorectal metastases the surgical margin can be, in particular situations, lowered to 2 mm with increase of the pathology recurrence rate from O% for 5 mm margin to 6% for 2 mm margin [107].

This finding, combined with a contrast-enhanced IOUS during the resection, could be a rationale incentive for practising limited resections [108–110], and the possibility of an accurate investigation of the remnant liver through the IOUS.

#### 2.1.14. Drug Administration for Reducing Intraoperative Blood Loss

Liver resection may cause a variable degree of hyperfibrinolytic states; this phenomenon occurs in the days immediately after hepatectomy and is more pronounced in patients with a diseased liver or in patients who have undergone to a wider hepatectomy extent [111–116]. So some authors propose the utilization of drugs with antifibrinolytic effect like Aprotinin that is reported to be capable of reducing intraoperative blood loss (especially during liver resection time) and transfusions [117–119]. Other authors propose utilization of the cheaper Tranexamic acid reporting similar results [120]. Although a theoretical risk of thromboembolic complications is present, no adverse drug effects like deep venous thrombosis, pulmonary embolism, or other circulatory disturbances were detected in both these studies.

## 3. Comparison of Different Liver Transection Techniques

The choice of transection techniques is currently a matter of preference for surgeons, as there are few data from prospective randomized trials that compared different techniques. It has been shown in small prospective randomized trials that clamp crushing or water jet may be preferable to CUSA in terms of quality of transection or speed of transaction [1, 121]. However, the results of these trials remain to be validated by larger-scale trials. CUSA dissection is still a widely used technique worldwide. Recently, a randomized trial compared four methods of liver transection, namely, clamp crushing, CUSA, Hydrojet, and dissecting sealer, with 25 patients in each group [122]. In that study, clamp crushing was associated with the fastest transection speed, lowest blood loss, and lowest blood transfusion requirement. Furthermore, clamp crushing was the most cost-effective technique. However, in that study, clamp crushing was performed with the Pringle maneuvre, whereas the other techniques were performed without the Pringle maneuvre. This might have resulted in bias in favor of clamp crushing. Another recent comparative study between clamp crushing technique (CRUSH), ultrasonic dissection (CUSA), or bipolar device (LigaSure) failed to show any difference between the three techniques in terms of intraoperative blood loss, blood transfusion, postoperative complications, and mortality [73]. Further prospective randomized studies are needed to determine which transection technique is the best. Moreover, a recent review of the Cochrane conclude that clamp-crush technique is advocated as the method of choice in liver parenchymal transection because it avoids special equipment, whereas the newer methods do not seem to offer any benefit in decreasing the morbidity or transfusion requirement. Otherwise in the comparison of different techniques, apart from the efficacy in transaction with low blood loss, the relative speed of transection and the potential complications are other parameters to be considered [121]. Furthermore, the use of special instruments for transection is costly, especially when two instruments are used in combination for transection and hemostasis. It is difficult to compare the relative cost of different transection instruments because some are reusable whereas others are designed for single use, and the cost of the same instrument varies substantially in different countries. Nonetheless, the cost of these various techniques should play a part in the surgeon's decision as to whether to use them or not.

### 3.1. The Role of the Anaesthetist

Patients who are subjected to liver surgery are usually pre- and intra-operationally treated with infusion of liquids, plasma expanders, and blood products: normally hepatic resections are in fact conduced in condition of euvolaemia or hypervolaemia to protect patients from the risk of consistent haemorrhage and haemodynamic's instability.

Despite this idea, several studies have demonstrated that a condition of low central venous pressure (LCVP) can reduce bleeding, recourse to vessel occlusion techniques, and transfusions during resection [111–113]. It has been scientifically demonstrated that intraoperative blood loss is correlated with inferior retrohepatic vena cava pressure [114].

Mendelez obtained very low blood loss results in major hepatic resections and managed to keep the CVP under 5 mmHg: this is possible with abstention from practising any infusion but intraoperative liquid infusion at the low speed of 75 mL/h and without any drug administration but employing hypotensive effects of normal anaesthetics (like Isoflurane, Morphine and Fentanyl). It is obvious that LCVP technique needs a strict monitoring of several parameters: in particular, systolic arterial pressure has constantly to be kept over 90 mmHg and diuresis over 25 mL/h. After the specimen is removed and after the realization of complete haemostasis starts, the infusion of liquids, and, if necessary, of plasma expanders and blood products until euvolaemia is obtained and haemoglobin value is over 8–10 g/dL [115].

LCVP has to be abandoned in case of uncontrollable haemorrhage (over 25% of total blood volume) or application of total vascular exclusion technique. Mendelez using LCVP reports a 0,4% rate of gas embolism [116]. This illustrates the importance of collaboration between surgeons and anaesthetists for a successful hepatectomy.

## 4. Conclusions

Improvement in the techniques of liver transection is one of the most important factors for improved safety of hepatectomy in recent years. The use of intraoperative ultrasound aids delineation of the proper transection plane and allows to transect tumor close to main vessels without bleeding. Clamp-crushing and ultrasonic dissection are currently the two most popular techniques of liver transection. The role of new instruments such as ultrasonic shear and RFA devices in liver transection remains unclear, with few data available in the literature.

The role of vascular exclusion including Pringle's maneuvre seems to be decreasing with improved transection technique. However, it remains a useful technique in reducing bleeding from inflow vessels, especially for surgeons with less experience in liver resection, and recent results show safety of this technique even for prolonged total time of ischemia. Maintenance of low central venous pressure remains an important adjunctive measure to reduce blood loss in liver transection.

As clear data for comparison of various liver transection techniques are lacking, currently the choice of technique is often based on the individual surgeon's preference. However, certain general recommendations can be made based on existing data and the author's experience. Clamp-crushing is a low-cost technique but it requires substantial experience to be used effectively for liver transection, especially in the cirrhotic liver. CUSA can be used in both cirrhotic and noncirrhotic liver, is associated with low blood loss, and has a well-established safety record, with low risk of bile leak. It is particularly useful in major hepatic resections when dissection of the major branches of the hepatic veins is required, or in cases where the tumor is in close proximity to a major hepatic vein, as it allows clear dissection of the hepatic vein from the tumor. The main disadvantage of the CUSA technique is slow transection.

Newer instruments such as the Harmonic scalpel, LigaSure, and TissueLink Dissector enhance the capability of hemostasis and allow faster transection. However, they lack the preciseness of CUSA in dissection of major hepatic veins, and HS more than others may be associated with increased risk of bile leak. Moreover, they are particularly useful in laparoscopic liver resection. They can also be used in combination with CUSA for sealing of vessels, but this increases the cost substantially. RFA-assisted transection is probably the most speedy liver transaction technique. However, the risk of thermal injury to major bile duct is a serious concern and its use is probably restricted to minor resection. Gyrus and Aquamantys are relatively new instruments and the literature does not allow to draw any conclusion about their efficacy and safety.

The experience of the surgeon in practising hepatic surgery, whatever is the method to perform it, is still a factor of primary importance. In spite of that, the advent of new diagnostic instruments, new devices for resection and coagulation, a better knowledge of the liver's anatomy and pathology, and a closer collaboration with the anaesthetist make the hepatic surgery a kind of surgery more defined and rational. From this point of view, new studies based on the use of different surgical strategies, association of different devices, and employment of different diagnostic and anaesthetic techniques are desirable.
